# Genetics, Epigenetics and Environmental Factors in Dental Development and Pathologies: Genes, Interactions and Dental Development

**DOI:** 10.3390/genes17101241

**Published:** 2026-10-08

**Authors:** Alan Henry Brook

**Affiliations:** 1 Adelaide Dental School, University of Adelaide, Adelaide, SA 5000, Australia; alan.brook@adelaide.edu.au; 2 Barts & London Dental Institute, Queen Mary University of London, London E1 2AD, UK

## 1. Welcome!

It is a pleasure and a privilege to present this Special Issue of *Genes*. This significant enterprise brings together 21 original, fully peer-reviewed papers from international colleagues, illustrating the dynamism of our research in the field of dental, oral and orofacial research.

This Special Issue has been designed to combine original research and comprehensive reviews. The collaboration provides a forum for the dissemination of current status updates and the identification of new research directions. I would like to express my sincere gratitude to all the contributing authors, the extensive panel of reviewers and the editorial staff at *Genes*.

## 2. Foundation

This Special Issue, titled “Genetics, Epigenetics and Environmental Factors in Dental Development and Pathologies: Genes, Interactions and Dental Development”, is the culmination of a 15-year series of conceptual developments.

A concept paper published in 2009 examined the interplay between genetic, environmental and epigenetic factors during the various stages of dental development. The study demonstrated that the emergence of the dentition, including its variations and anomalies, is a complex process influenced by multiple factors at various levels, across both space and time [1]. In 2011, the development of the dentition was demonstrated to satisfy the core principles of a Complex Adaptive System on an international scale for the first time [2]. This concept was further advanced, demonstrating that general and craniofacial development are Complex Adaptive Systems. These systems are influenced by diversity, and it has been determined that multiple interacting factors during dental morphogenesis produce variations in tooth number, size, and shape [3,4].

Further advances revealed that the interactions of environmental stress and gene expression during dental development, as well as the changes in the mature dentition over time, are also CAS components [5,6]. In 2023, the concept of Many Model Thinking was integrated into the existing frameworks of Complexity and Networking. The development of mature dentition is influenced by a Multi-level Complex Interactive Network, and no single aetiological model can fully capture all aspects of the developmental process; rather, different models offer complementary insights [7].

Against this background, the papers in this Special Issue express the flow of dental development and pathologies of adult dental disease.

## 3. Fundamental Aspects of Craniofacial Development

The first section, papers 1 to 3, address fundamental aspects of craniofacial development and how it can be further investigated to enhance understanding and lay the foundations for future clinical advances. Spatial Transcriptomics is set to make significant advances in the field of temporal and spatial gene expression profiles, with the potential to identify novel therapeutic targets for the treatment of craniofacial disorders. Computational mapping will enhance our knowledge of molecular and cellular processes, while Tooth Formation is a valuable experimental model for understanding the dosage effects of chemotherapeutic agents.

## 4. Tooth Number—Tooth Agenesis/Supernumerary/Tooth Morphology

During the Initiation and Morphogenetic phases of tooth development, the number of teeth in a dentition is determined, as are their size and shape. The process is iterative and interactive between agents in the epithelial and ectomesenchyme. It is important to note that some of the genes in question act repeatedly during these stages, and these interactions emerge not only as Tooth Agenesis and Supernumerary teeth but also as variations in tooth morphology.

## 5. Enamel—Amelogenesis Imperfecta

As tooth development progresses, with the differentiation of the tissues in the tooth germ and mineralisation, the forming enamel is influenced by different genes, epigenetic and environmental effects.

Paper 11 provides a comprehensive review of enamel phenotypes, along with an analysis of their genetic and environmental determinants. This is followed by papers introducing a novel diagnostic approach in Amelogenesis Imperfecta and novel mutations in its aetiology.

## 6. Eruption

The eruption process comprises several interacting components. The emergence of teeth is governed by crucial genetic factors, with mutations in these genes potentially resulting in dental eruption failure, as outlined in papers 14 and 15.

## 7. Caries, Periodontal Disease—Apical Periodontitis

The erupted mature teeth and supporting tissues are subject to pathological changes. While the environmental agents involved have received significant attention, research is now focusing on the role of genetics and the interactions between genetic, epigenetic and environmental factors in caries, periodontal diseases and apical periodontitis. Papers 16, 17, 18, and 19 contributed to this research.

## 8. Teaching of Genetics

Genetics is an essential component of the education of all members of the Dental Team due to its role in normal and abnormal development, and in oral pathology. Such teaching must be targeted and tailored for dental students, employing the latest educational developments. Papers 20 and 21 address this need, arising from a Scandinavian initiative on which I am honoured to serve as an external advisor.

It is my hope that the papers in this volume will provide you with the same level of enjoyment and intellectual stimulation that I have experienced in my role as Guest Editor for this Special Issue of *Genes*.
